# Supplementary material for: How Healthcare Teams Appear to the Observer—An Approach to the Complexities of Mask-Wearing Attitudes During the COVID-19 Pandemic
Source: Int J Environ Res Public Health. 2025 Nov 20;22(11):1755. doi: 10.3390/ijerph22111755 (PMC12652415; doi:10.3390/ijerph22111755)
Supplement: Supplementary file 1 [file ijerph-22-01755-s001.zip › ijerph-3915529-supplementary.pdf]

Appendix I to

Visual assessment of healthcare teams – An approach to the complexities of mask wearing attitudes during the Covid-19 pandemic

Questionnaire

Dear Sir or Madam,

we would like to cordially invite you to participate in our survey on the above topic and kindly ask you to answer the questions in the following questionnaire.

We are investigating the influence of visual impressions on our perception of competence, likeability and trust. These vary in social perception and are subject to temporal fluctuations. With this study we want to investigate how we deal with the different forms of presentation nowadays.

This survey is being conducted as part of Hans-Joachim Röhrens' dissertation together with Prof. Dr. Michaela Zupanic and Prof. Dr. Jan P. Ehlers from the Department of Didactics and Educational Research at the University of Witten/ Herdecke. We are happy to try to answer the important questions here at the beginning:

Who can participate? Anyone over the age of 18, since we are all patients.

Extent? The online survey takes about 20 minutes.

Anonymity? Absolutely. For more information, please see the privacy policy.

Data protection information according to Art. 13 DSGVO:

This online survey of the UW/H is conducted at the Chair of Didactics and Educational Research in Health Care, Faculty of Health. The responsibility for the content lies with Prof. Dr. Jan Ehlers (jan.ehlers@uni-wh.de), Alfred-Herrhausen-Str. 50, 58448 Witten.

The processing of the survey data is solely for the purpose of scientific research. The study title is: What effect does the clothing of interprofessional teams have on the feelings of patients in an international comparison?

Only the information that you provide directly to us via the fields of the online survey will be included in the research dataset. Connection data that are additionally transmitted by your terminal device in the background for technical reasons are stored separately from the research data and only processed for a short period of time (e.g., for purposes of system maintenance and troubleshooting). This data is generally excluded from processing for research purposes.

Survey data is only kept for as long as necessary to fulfill the specific research purpose and according to legal requirements for research projects. The data will be analyzed anonymously on a comparative basis. Your responses will also be used to create non-identifiable information that we use both

individually and in aggregate with information from other sources. No further processing of personal data or transfer to third parties outside the UW/H is intended. Your data will not be disclosed except in an aggregate or unidentified form.

The legal basis for the processing of the research data is your voluntary consent according to Art. 6 (1) a DSGVO, which you actively express by clicking the button below and calling up the first page of the online survey. UW/H processes the additional background data in accordance with Art. 6 (1) f DSGVO in its own legitimate interest, but exclusively for the purpose of maintaining its web systems.

The granting of your consent is voluntary and anonymous. You can cancel the survey at any time.

Further general information on UW/H data protection can be found at https://www.uni-wh.de/datenschutz/. There, in addition to additional information on data subject rights, you will also find detailed information on the connection data that is transmitted to the UW/H in the background together with the survey data.

You can reach the UW/H data protection officer at datenschutz@uni-wh.de.

You have the right to complain to the data protection authority of the country in which you live or work or in which you suspect a violation of data protection law.

I agree to the data protection notice and the processing of my data and would like to participate in the survey

□ Yes (-> Participation in survey)

□ No (-> Forwarding to www.uni-wh.de)

Sociodemographic data

- How old are you? ___ (pull-down menu, 18-99 years)

- What is your gender? □ diverse □ female □ male

- What is your nationality? (pulldown menu, nationalities)

- What is your highest level of education? (Pulldown menu: none, Hauptschule, Realschule, Abitur, vocational training, university degree, doctorate, habilitation)

- In which field do you work? (pulldown menu, fields of work + other with free text)

- How much does the CoVid19 pandemic affect you? (Likert 1-6, from not at all to very much)

- How do you rate the Corona measures in your country (Likert 1-9, way too lax - just right - completely over the top).

Picture 1

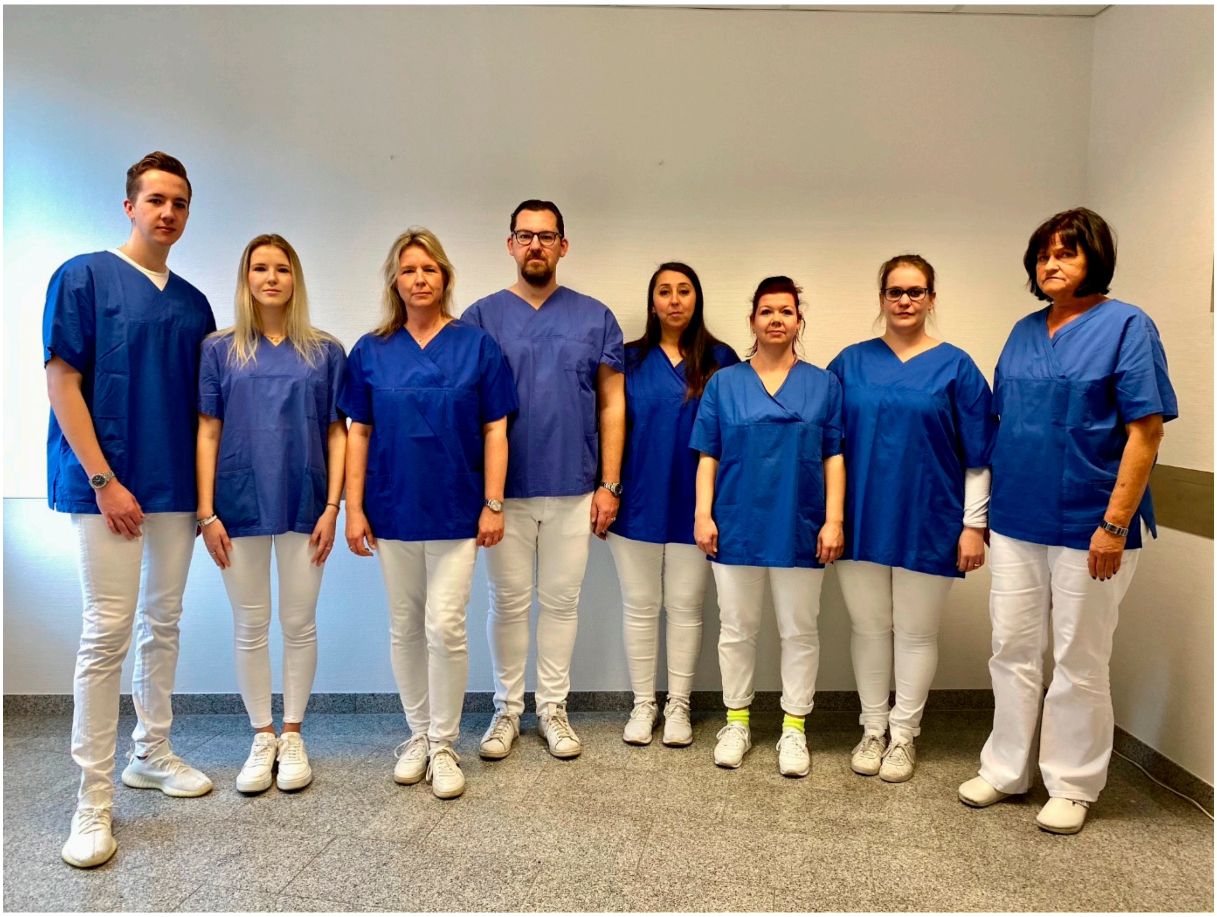

Please assign the persons their professions from left to right. (Persons are marked with numbers in the questionnaire)

1st person (pulldown: physician, medical assistant, diabetes assistant, trainee medical assistant, medical student, relieving care assistant)

2nd person (pulldown: physician, medical assistant, diabetes assistant, trainee medical assistant, medical student, relieving care assistant)

3rd person (pulldown: physician, medical assistant, diabetes assistant, trainee medical assistant, medical student, relieving care assistant)

4th person (pulldown: physician, medical assistant, diabetes assistant, trainee medical assistant, medical student, relieving care assistant)

5th person (pulldown: physician, medical assistant, diabetes assistant, trainee medical assistant, medical student, relieving care assistant)

6th person (pulldown: physician, medical assistant, diabetes assistant, trainee medical assistant, medical student, relieving care assistant)

7th person (pulldown: physician, medical assistant, diabetes assistant, trainee medical assistant, medical student, relieving care assistant)

8th person (pulldown: physician, medical assistant, diabetes assistant, trainee medical assistant, medical student, relieving care assistant)

How likeable is this practice team to you? (1= not at all - 6 very likeable)

How competent does this practice team seem to you? (1= not at all - 6 very competent)

How much would you trust this practice team? (1= not at all - 6 very much)

Would you choose this practice as your primary care practice? (1= no, never - 6 yes, definitely)

What would you use to visit this practice? (Multiple choice)

□ Routine examination

□ minor illness

□ severe illness

□ chronic illness

□ mental illness

□ not at all

Would you want to work on this team? (1= no, never - 6 yes, definitely).

Picture 2

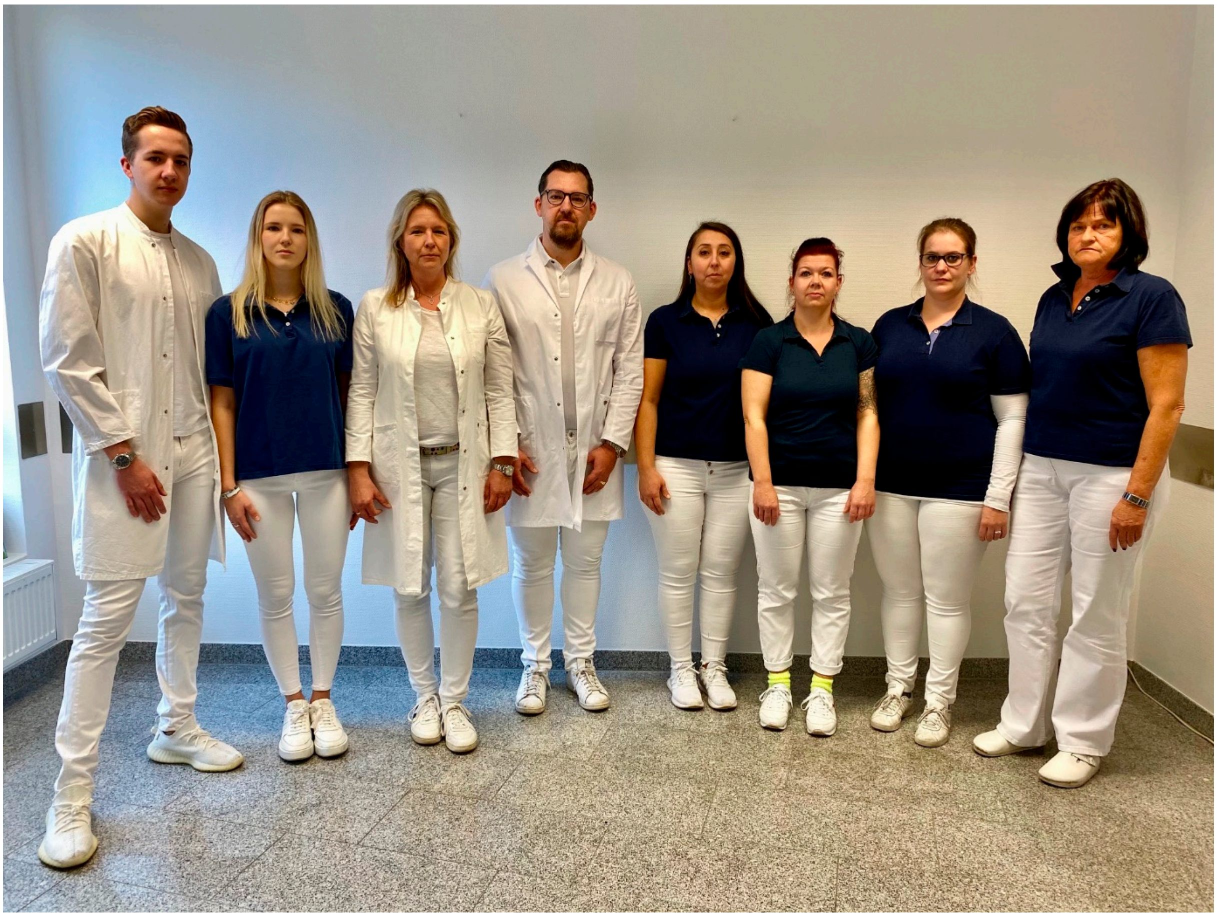

Please assign the persons their professions from left to right. (Persons are marked with numbers in the questionnaire)

1st person (pulldown: physician, medical assistant, diabetes assistant, trainee medical assistant, medical student, relieving care assistant)

2nd person (pulldown: physician, medical assistant, diabetes assistant, trainee medical assistant, medical student, relieving care assistant)

3rd person (pulldown: physician, medical assistant, diabetes assistant, trainee medical assistant, medical student, relieving care assistant)

4th person (pulldown: physician, medical assistant, diabetes assistant, trainee medical assistant, medical student, relieving care assistant)

5th person (pulldown: physician, medical assistant, diabetes assistant, trainee medical assistant, medical student, relieving care assistant)

6th person (pulldown: physician, medical assistant, diabetes assistant, trainee medical assistant, medical student, relieving care assistant)

7th person (pulldown: physician, medical assistant, diabetes assistant, trainee medical assistant, medical student, relieving care assistant)

8th person (pulldown: physician, medical assistant, diabetes assistant, trainee medical assistant, medical student, relieving care assistant)

How likeable is this practice team to you? (1= not at all - 6 very likeable)

How competent does this practice team seem to you? (1= not at all - 6 very competent)

How much would you trust this practice team? (1= not at all - 6 very much)

Would you choose this practice as your primary care practice? (1= no, never - 6 yes, definitely)

What would you use to visit this practice? (Multiple choice)

□ Routine examination

□ minor illness

□ severe illness

□ chronic illness

□ mental illness

□ not at all

Would you want to work on this team? (1= no, never - 6 yes, definitely).

Picture 3

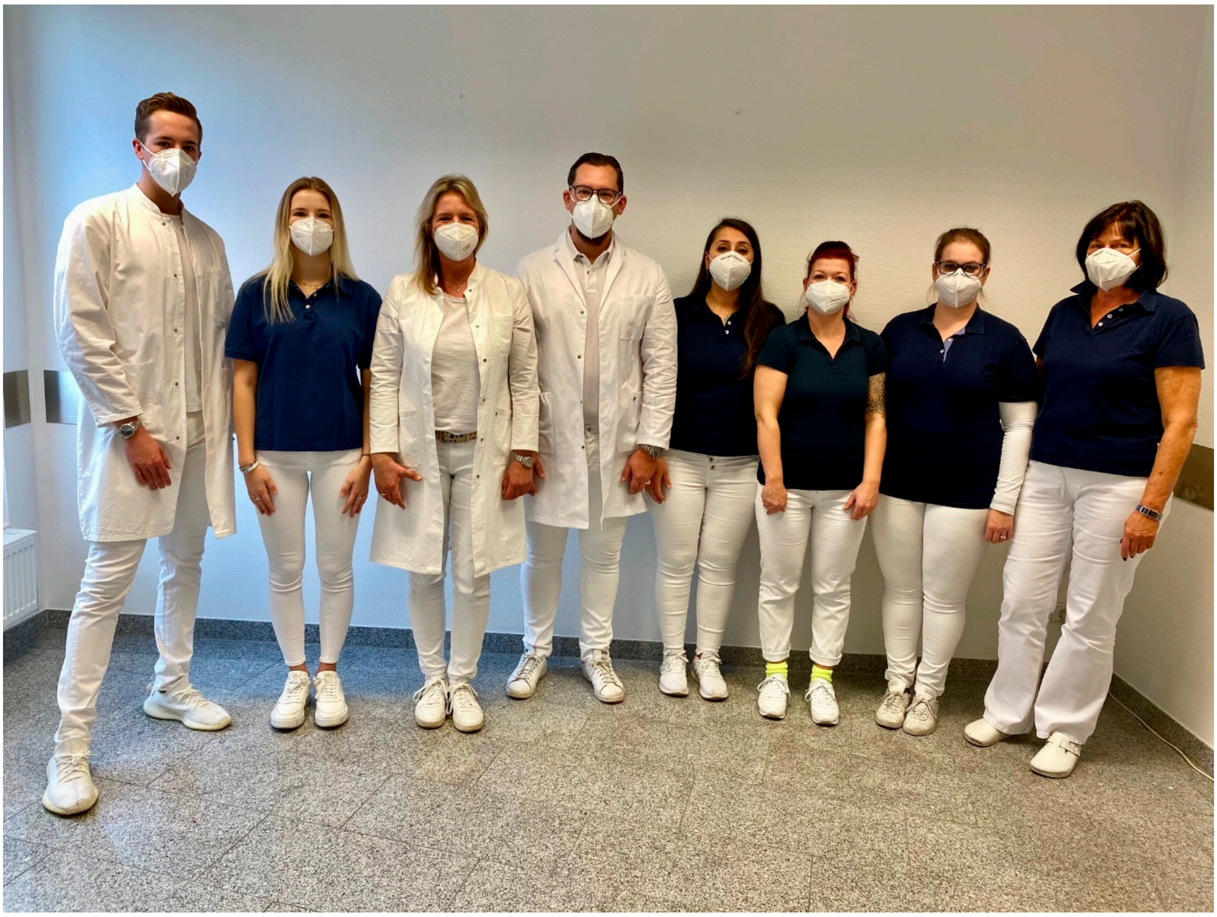

How likeable is this practice team to you? (1= not at all - 6 very likeable)

How competent does this practice team seem to you? (1= not at all - 6 very competent)

How much would you trust this practice team? (1= not at all - 6 very much)

Would you choose this practice as your primary care practice? (1= no, never - 6 yes, definitely)

What would you use to visit this practice? (Multiple choice)

□ Routine examination

□ minor illness

□ severe illness

□ chronic illness

□ mental illness

□ not at all

Would you want to work on this team? (1= no, never - 6 yes, definitely).

Picture 4

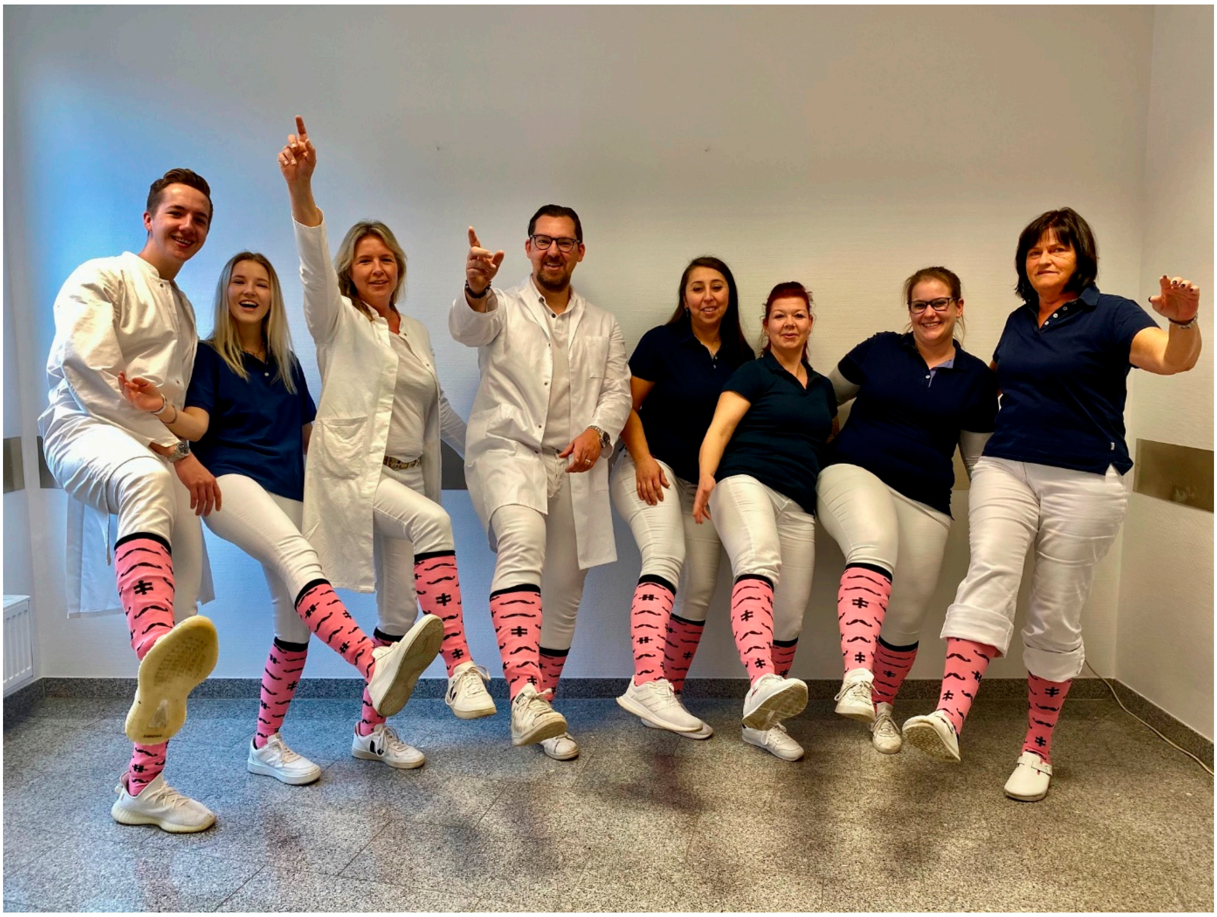

How likeable is this practice team to you? (1= not at all - 6 very likeable)

How competent does this practice team seem to you? (1= not at all - 6 very competent)

How much would you trust this practice team? (1= not at all - 6 very much)

Would you choose this practice as your primary care practice? (1= no, never - 6 yes, definitely)

What would you use to visit this practice? (Multiple choice)

□ Routine examination

□ minor illness

□ severe illness

□ chronic illness

□ mental illness

□ not at all

Would you want to work on this team? (1= no, never - 6 yes, definitely).

Thank you very much for participating in our survey! Stay healthy!
